# Morphological Differences between β_2_-Microglobulin in Fibrils and Inclusion Bodies

**DOI:** 10.1002/cbic.201000582

**Published:** 2011-01-26

**Authors:** Garrick F Taylor, Stephen P Wood, Karsten Mörs, Clemens Glaubitz, Jörn M Werner, Philip T F Williamson

**Affiliations:** [a] School of Biological Sciences, University of SouthamptonHighfield Campus, Southampton, SO17 1BJ (UK); [b] Centre for Amyloidosis and Acute Phase Proteins, Division of Medicine (Royal Free Campus), University College London Medical SchoolRowland Hill Street, London NW3 2PF (UK); [c] Goethe UniversitätMax von Laue Strasse 9, 60438 Frankfurt am Main (Germany)

**Keywords:** amyloid fibrils, inclusion bodies, microglobulin, protein folding, solid-state NMR spectroscopy

The over-expression of recombinant protein in *E. coli* frequently results in the formation of insoluble aggregates, commonly referred to as inclusion bodies (IBs). It was widely believed that within the IBs the proteins were misfolded and functionally inactive, until a number of studies revealed that for some proteins there is at least a population within the IBs that are functionally competent or contain functionally competent domains[1] and structurally native folds.[2] More-recent studies have focused on the similarities between IBs and amyloid fibrils,[3] such as those found in a number of diseases including localized conditions such as Alzheimer's and Huntington's as well as systemic conditions including reactive systemic amyloidosis and dialysis-related amyloidosis.[4] Interestingly, these studies have revealed that, upon the expression of some proteins in *E. coli*, the IBs formed possess many of the properties associated with amyloid deposits, including the characteristic spectral changes associated with the binding of the dyes Congo Red and Thioflavin T (ThT).[4] Similarly, structural studies reveal the presence of cross-β structures[3a] and a local molecular geometry identical to that of fibrils.[5] These observations have led to the speculation that IBs and amyloid deposits could share a common functional role, namely the sequestration of misfolded protein to prevent damage to the host. Here, we report on studies of IBs composed of β_2_-microglobulin (β_2_m) that are formed upon expression in *E. coli* and compare their amyloid properties with those of β_2_m fibrils similar to those found in patients suffering from dialysis-related amyloidosis.

The fluorescence emission spectrum of ThT is widely used as an indicator for the formation of amyloid fibrils.[6] To ascertain whether β_2_m IBs possess the necessary structural properties to bind ThT, emission spectra were recorded in the presence of IBs, β_2_m fibrils created by acid precipitation and soluble β_2_m (Figure 1). Both the β_2_m fibrils and IBs showed enhanced fluorescence at 485 nm compared with the soluble β_2_m. Interestingly, ThT fluorescence for the IBs was only 11% of that of the fibrillar material. This suggests that, within the IBs, either only a fraction of the protein exists in a fibrillar form capable of enhancing fluorescence or that the β_2_m adopts a conformation that results in a lower enhancement of fluorescence than when bound to fibrillar structures.

**Figure 1:**
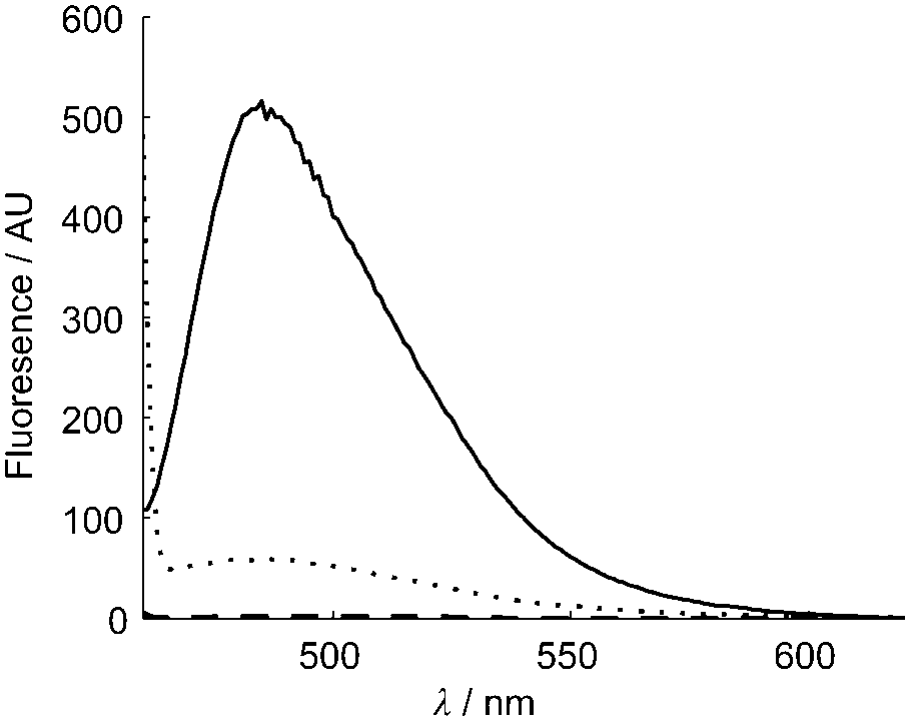
ThT emission spectra in 1 mg mL^−1^ solutions of β_2_m fibrils (—), inclusion bodies (…) and monomeric β_2_m (- - - -). ThT was excited at a wavelength of 440 nm.

To characterize the differences between the IBs and fibrillar β_2_m at the molecular level, magic-angle spinning (MAS) solid-state NMR measurements were performed. Using ^13^C/^13^C two-dimensional proton-driven spin diffusion (PDSD) MAS NMR spectroscopy,[7] we were able to map out connectivities between proximal ^13^C atoms. This allowed for resonances to be assigned to particular sites within the amino acids of β_2_m in fibrils and IBs on the basis of published random-coil chemical shifts obtained in solution[8] (Figures S2 and S3) and provided insights into the local conformation and electrostatic environment.

The PDSD ^13^C/^13^C correlation spectrum of the fibrillar β_2_m (Figure 2, blue contours) shows line widths of ∼0.5 ppm, which are comparable with those observed for other ^13^C/^15^N-labeled fibrillar systems.[9] As expected for the short mixing times employed, the spectrum is dominated by correlations between adjacent carbon atoms and other intraresidue transfers. The spectrum is relatively crowded due to the overall size of the protein; however, the resonances are sufficiently well resolved to be assigned to specific amino acid types and, in favorable cases, to particular sites within the protein. These assignments are consistent with a recently published partial assignment of long, straight form of fibrillar β_2_m in the solid state.[10]

**Figure 2:**
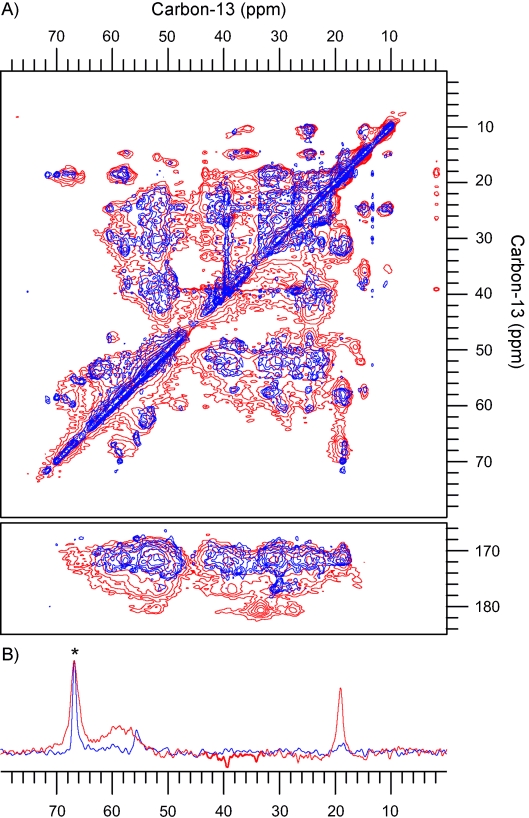
A) Expansion of the 100 ms proton-driven spin-diffusion homonuclear ^13^C/^13^C correlation spectra of β_2_m acid produced fibrils (blue) and IBs (red). Spectra were recorded at 5 °C with a MAS spinning frequency of 15 kHz and plotted with the lowest contour just above the noise threshold and subsequent levels at multiples of 1.625. (Non-superimposed spectra are given in the Supporting Information.) B) 1D slice through the 2D spectrum at 67 ppm showing differences in line width and resonance positions of the fibrils and IBs, the diagonal peak is indicated by an asterisk.

The spectrum of β_2_m IBs (Figure 2 A, red contours) is dominated by intense protein resonances that arise from β_2_m within the IBs. There is no evidence of other molecular species such as oligosaccharides, DNA, or lipids, as previously observed in IB preparations of other systems.[5] The lack of resonances from these species in direct excitation spectra (data not shown) indicates that this is due to their absence not just the selective observation of immobile components by crosspolarization. The spectra of IBs are again dominated by intraresidue crosspeaks; however, these are significantly more intense than the fibrillar β_2_m peaks. The efficient proton driven spin diffusion and crosspolarization throughout the spectra suggests that the dipolar couplings within the IB sample are stronger than those in the fibrillar material, and this implies that the protein exhibits less mobility. This efficient proton-driven spin diffusion also leads to a number of additional crosspeaks within the IB spectrum compared to the fibrillar material. In particular, significant intensity is apparent at sites that correspond to correlations between sites in the side chains containing carboxylic/carboxamide groups (Asp/Glu/Asn/Gln) at 58–25 and 175–185 ppm and correlations between the aromatic side chains (Phe/Tyr) and the corresponding β-carbon atoms (∼130, 30 ppm; Figure S3).[8] In addition, correlations between resonances attributed to aliphatic side chains, in particular Ile, show significantly enhanced intensity.

The resonances observed in the IB spectrum are significantly broader than those observed in the fibrillar spectrum. We attribute this increase to the lower mobility within the IBs, which results in an increase in the homogeneous line width. This is consistent with the enhanced spin diffusion observed throughout the PDSD spectra of the IBs. Based on the earlier perception that IBs are composed of misfolded protein, it was expected that significant inhomogeneous broadening would have been observed due to the structural heterogeneities within the sample. However, resonances can be resolved in the IB spectra with line widths of 1.0 ppm, similar to those observed in other noncrystalline, yet structurally homogeneous fibrillar systems.[9] The observed increase in homogeneous line width is further supported by inspection of the resonances along the diagonal. It is expected that inhomogeneous broadening would lead to a distribution of resonances along the diagonal, whilst an increase in the homogeneous line width would result in a broadening of the diagonal. Comparison of the diagonal resonances in the fibrillar and IB spectra shows a clear increase in line width from ∼0.5 to 1.2 ppm (Figure 2 B); this is consistent with an increase in homogeneous line width.

The enhanced crosspeak intensity and larger line width observed in the IB spectrum suggest significant dynamic differences between the fibrillar and IB forms of β_2_m. In addition, there are noticeable differences in the positions of a number of resonances, thus suggesting differences in local structure or environments. These could be responsible for the lower ThT fluorescence that is observed upon the binding of ThT to the IBs.

In summary, our studies of β_2_m fibrils and inclusion bodies show marked differences both in their response to ThT binding and, at a molecular level, their structure and dynamics, as revealed by MAS NMR spectroscopy. These findings contrast with recent studies of IBs composed of the fungal prion protein Het-S, a protein with an intrinsic propensity to form amyloid fibrils. Solid-state NMR studies of Het-S IBs have indicated that, at a molecular level, the protein adopts a conformation identical to that of the fibrillar Het-S with little structural heterogeneity whilst electron-microscopy and dye-binding assays confirm the fibrillar nature of the material.[5]

The observations on β_2_m suggest that formation of fibrillar structures is not the default aggregation pathway and that other outcomes are possible. Indeed, it is widely acknowledged from in vitro experiments[11] that β_2_m requires external factors to promote fibril formation. This leads us to suggest that the propensity of a protein to adopt a fibrillar structure whilst being expressed in *E. coli* reflects an individual protein's intrinsic potential to adopt a conformation that promotes fibrillogenesis. Our observations contrast with earlier molecular studies of IBs[3a, 5, 12] that were conducted on proteins known to readily adopt a fibrillar structure and thus can be expected to form fibrils following expression. This leads us to conclude that during expression not all proteins adopt the necessary intermediate that promotes fibrillogenesis, but instead might form states that favor highly dense aggregates composed of structured protein.

## Experimental Section

**Preparation of β_2_m inclusion bodies and monomeric β_2_m:** The β_2_m labeled with ^13^C and ^15^N was expressed to inclusion bodies in BL21(DE3) *E. coli* transformed with a pET11a plasmid encoding the human form of β_2_m. The bacteria were grown on M9 salts (1 L) containing uniformly labeled [^13^C]glucose and [^15^N]ammonium chloride (Cambridge Isotopes Ltd.) until an OD_600_ of 1.0 was reached, at which point β_2_m expression was induced by the addition of isopropyl-β-d-thiogalactopyranoside to a final concentration of 1 mm. After a further 16 h, the bacteria were harvested by centrifugation (8983 *g*, 20 min). The bacteria were broken by using a probe sonicator (10 min), and the insoluble material was pelleted by centrifugation (16 000 *g*, 20 min). The pellet was washed with wash buffer (2×25 mL; 50 mm TRIS, 100 mm NaCl, 4.2 mm MgCl, 0.5% Triton-X100, pH 8.0) containing lysozyme and DNaseI (Sigma). The pellet was subjected to a further five washes with wash buffer to give the final inclusion body sample.

To regenerate monomeric β_2_m fibril formation, the inclusion bodies were homogenized in solubilization buffer (8 m urea, 50 mm 2-morpholinoethane sulfonic acid, 0.1 mm ethylenediaminetetraacetate (EDTA), 0.2 mm dithiothreitol), stirred overnight, and clarified by centrifugation. The solubilized β_2_m was refolded by slow dilution into arginine buffer (100 mm TRIS, 400 mm l-arginine, 2 mm EDTA, 5 mm reduced glutathione, 0.5 mm oxidized glutathione, 0.1 mm phenylmethylsulfonyl fluoride), and the solution was incubated for a further 12 h. The diluted protein was concentrated by using Kvick cross filtration equipment with cellulose membranes and with a 5000 kDa molecular weight cut off (GE Healthcare) and the monomeric form purified by using a Superdex 75-filled Hiload 16/66 size-exclusion column (Amersham Biosciences) equilibrated with HEPES buffer (25 mm HEPES, 50 mm KCl, 0.1% NaN_3,_ pH 7.4). The monomeric form was concentrated to final stock concentration by using Vivaspin Ultrafiltration devices (Sartorious, Epsom, UK). The concentration of the protein samples was determined spectrophotometrically to be 280 nm (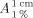 = 16.91). The purity of both fibrils and inclusion bodies was confirmed by SDS-PAGE following solubilization in urea (Figure S1).

**Formation of β_2_m fibrils:** Fibrils composed of β_2_m were prepared by acid precipitation. Monomeric β_2_m (84 μm β_2_m, 25 mm HEPES, 50 mm KCl, pH 7.4) was mixed 1:1 with a low-pH sodium citrate buffer (50 mm sodium citrate, pH 1.6) to a final pH of 2.5. The solution was incubated at 37 °C with 200 rpm orbital shaking for 1 week. Fibril formation was confirmed by a Congo Red spectrophotometric assay that showed a characteristic shift in absorbance from 403 to 541 nm (data not shown). Fibril formation was also determined by a Thioflavin T fluorescence assay, with *λ*_ex_=420 nm by monitoring the increase in emission at 485 nm upon fibril formation. Analysis of the fibrillar material by negative-stain electron microscopy revealed extended fibrillar structures consistent with previous studies. Prior to NMR analysis, the β_2_m fibrils were harvested by ultracentrifugation (98 400 *g*, 60 min).

**Solid-state NMR studies:** Spectra were acquired on an 850 mhz Avance Spectrometer (Bruker) equipped with a 3.2 mm triple resonance CP-MAS probe. Typically the β_2_m inclusion bodies and fibrils were composed of approximately 50–70% water *w*/*w* as determined by lyophilization; this permitted the packing of 10 mg of protein into a 3.2 mm rotor for NMR analysis. Data were acquired with 15 kHz MAS with a sample temperature of approximately 5 °C (temperature set to −10 °C to compensate for frictional and radio frequency (rf) heating). ^13^C homonuclear correlation spectra were acquired on a standard proton-driven spin diffusion (100 ms) exchange experiment.[7] Carbon magnetization was excited by adiabatic cross polarization with a carbon rf amplitude centered on 75 kHz. Two-dimensional spectra were acquired phase sensitively with a time-proportional phase increment (TPPI) in the indirect dimension and with a 65 kHz spectral width in each dimension. A total 600 *t*_1_ increments were acquired in the indirect dimension with 64 scans per increment and a 2.5 s recycle delay. During both direct and indirect dimensions SPINAL proton decoupled was applied with an rf amplitude of 120 kHz. The data were processed in nmrPipe[13] and analysed in CCPN.[14] A sine-bell-squared function was applied to both dimensions prior to zero filling to 4096 points in each dimension and Fourier transformation.
