# Supplementary material for: Morphological Differences between β2-Microglobulin in Fibrils and Inclusion Bodies
Source: Chembiochem. 2011 Jan 26;12(4):556–8. doi: 10.1002/cbic.201000582 (PMC3084992; doi:10.1002/cbic.201000582)
Supplement: Supplementary file 1 [file cbic0012-0556-SD1.pdf]

CHEMBIOCHEM

Supporting Information

© Copyright Wiley-VCH Verlag GmbH & Co. KGaA, 69451 Weinheim, 2011

Morphological Differences between β_2-Microglobulin in Fibrils and Inclusion Bodies

Garrick F. Taylor,[a] Stephen P. Wood,[b] Karsten Mörs,[c] Clemens Glaubitz,[c] Jörn M. Werner,[a] and Philip T. F. Williamson*[a]

cbic_201000582_sm_miscellaneous_information.pdf

A. Purification of β_2-Microglobulin

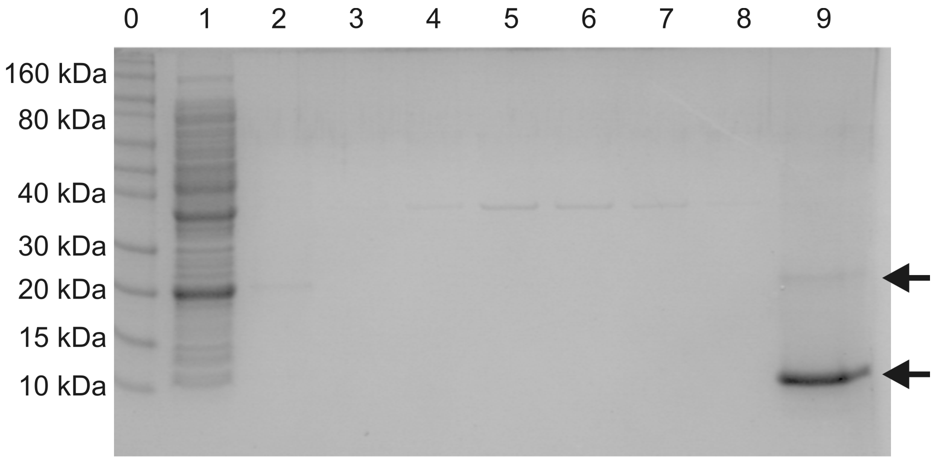

Figure S1. SDS-PAGE (10%) showing the purification and solubilization of β_2-microglobulin. Lane 0, molecular weight markers. Lanes 1-8, supernatant from Triton-X100 washes of the β_2-microglobulin inclusion bodies. Lane 9, urea solubilized β_2-microglobulin with bands corresponding to the monomeric and dimeric form (indicated by arrows).

B. Proton Driven Spin Diffusion Spectra of β_2-Microglobulin Inclusion Bodies and Fibrils.

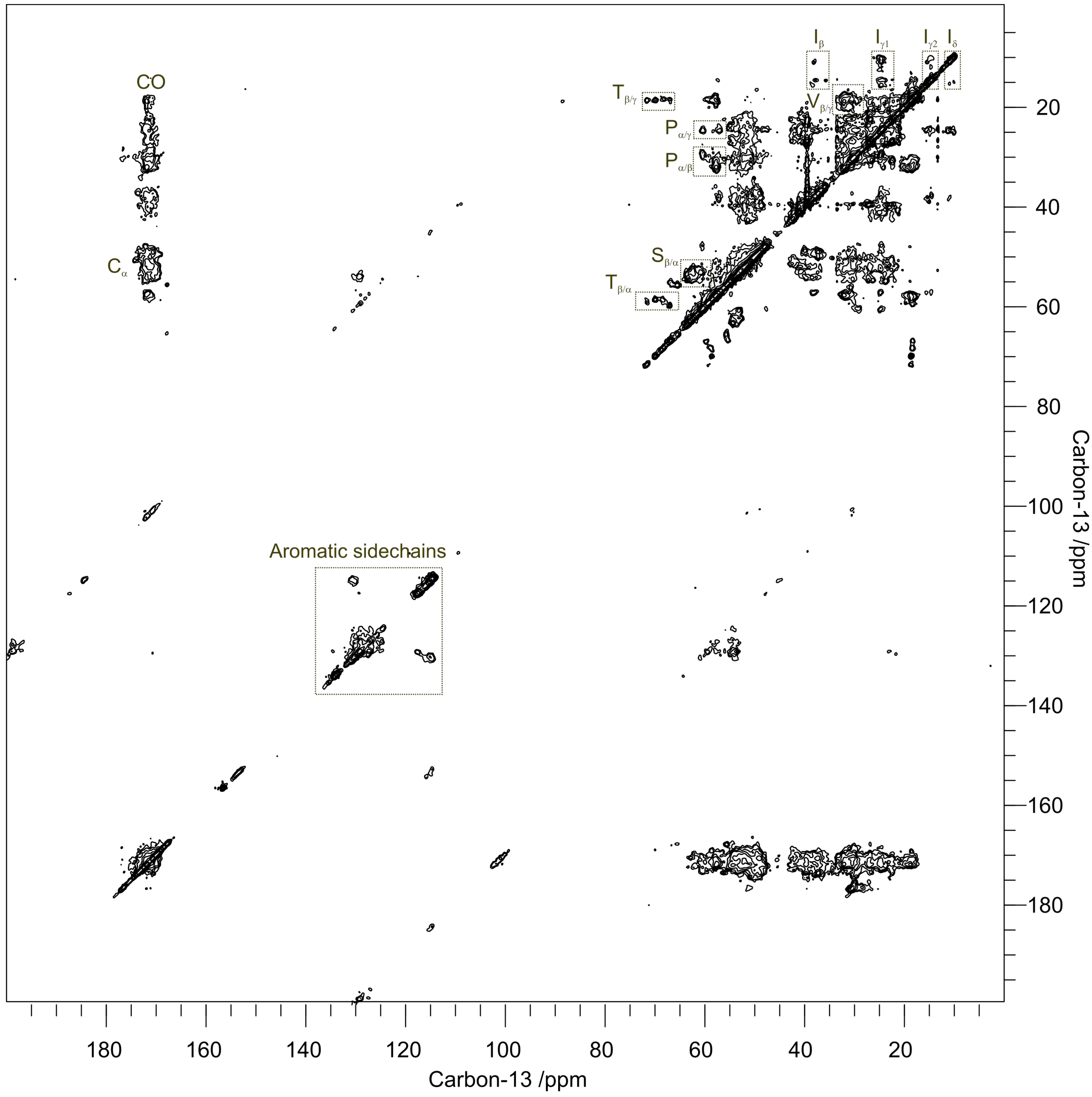

Figure S2. Proton driven spin diffusion spectrum of β_2-microglobulin fibrils produced by acid precipitation. Assignments made on the basis of distinct random coil chemical shifts and observed correlations. As expected for the short mixing time employed (100ms) the correlations observed arise primarily from intra-residue magnetization exchange.

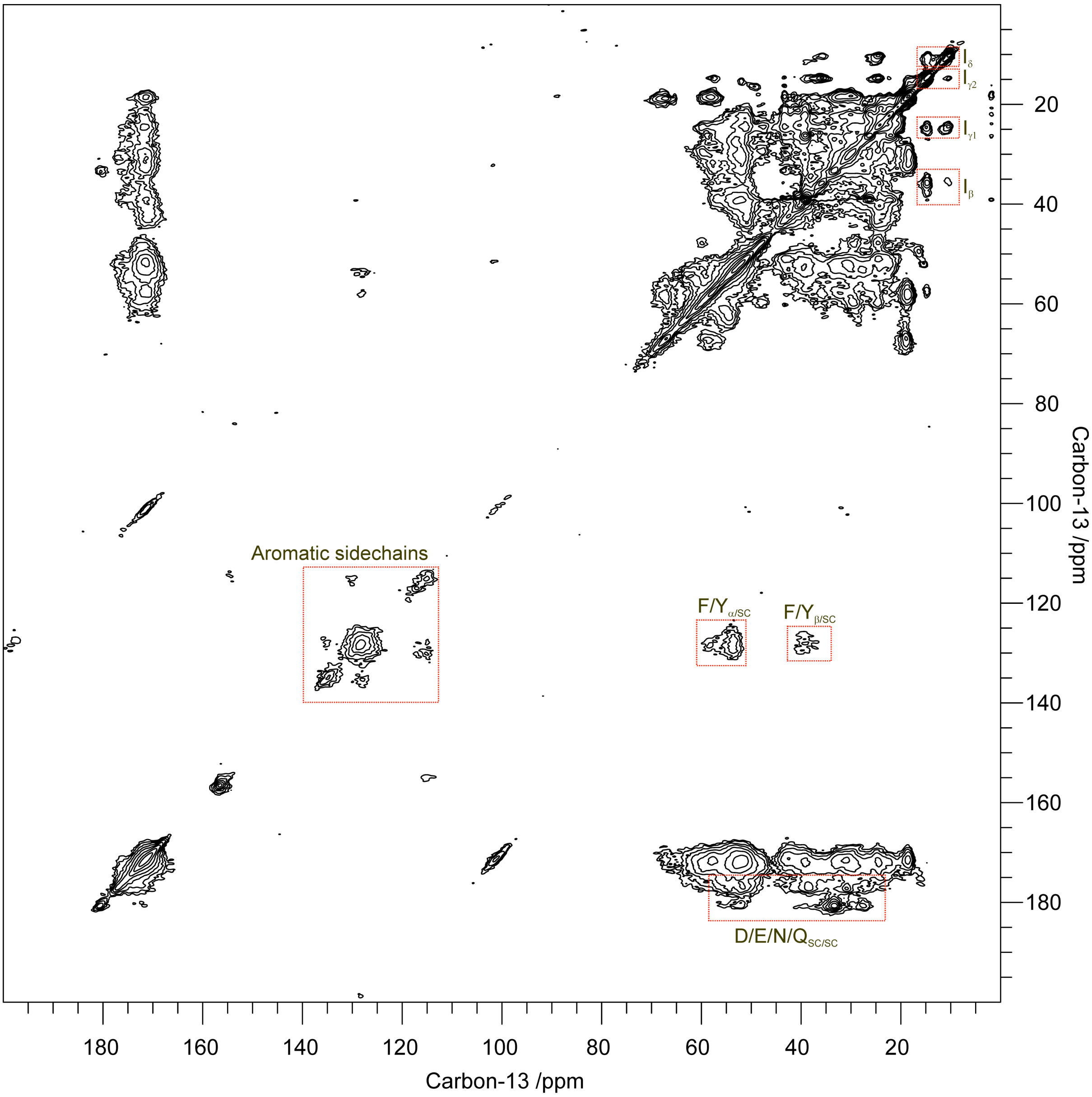

Figure S3. Proton driven spin diffusion spectrum of β_2-microglobulin inclusion bodies. The assignable sites showing more intense cross peaks discussed in the main paper are highlighted in red boxes. The short mixing time again leads to a spectrum dominated by correlations arising from intra-residue magnetization exchange.
